# Intravenous Efgartigimod Alfa as Initial Monotherapy for Disabling Ocular Myasthenia in an Elderly Patient with Multiple Comorbidities

**DOI:** 10.7759/cureus.74768

**Published:** 2024-11-29

**Authors:** Karen Inzirillo, Octavio Carranza, Marc A Swerdloff

**Affiliations:** 1 Neurology, Marcus Neuroscience Institute, Boca Raton Regional Hospital, Baptist Health South Florida, Boca Raton, USA; 2 Neurology, Charles E. Schmidt College of Medicine, Florida Atlantic University, Boca Raton, USA

**Keywords:** autoimmune disease, efgartigimod alfa, ocular myasthenia, older population, positive antibodies, ptosis

## Abstract

Myasthenia gravis (MG) is one of the most common neuromuscular disorders. It is an antibody-mediated autoimmune disease affecting the neuromuscular junction, presenting with fluctuating muscle weakness that commonly affects the ocular, bulbar, proximal, and respiratory muscles. Treating MG in the older population with preexisting comorbidities can be challenging. Intravenous efgartigimod alfa (EA) was successfully used as an initial monotherapy for a 90-year-old woman with acetylcholine receptor seropositive MG and stage IV colon adenocarcinoma who was referred for visually disabling bilateral eyelid ptosis. Standard MG therapy was considered but not chosen due to relative contraindications and anticipated adverse effects. EA infusions were well tolerated and corrected her ptosis after two weeks of treatment. EA infusion may be considered the first-line therapy for selected patients with seropositive generalized MG who have disabling ocular symptoms.

## Introduction

Myasthenia gravis (MG) is a chronic autoimmune disorder that targets the neuromuscular junction, resulting in variable muscle weakness. According to the Myasthenia Gravis Foundation of America, the prevalence is 14-20 per 100,000, affecting up to 60,000 individuals in the United States [1]. It is the most common treatable neuromuscular junction disorder [1,2]. Unfortunately, up to 13% of MG patients are refractory or intolerant to therapy [2,3]. The age of 50 divides early-onset MG (EOMG) from late-onset MG (LOMG), with EOMG being more frequent in women and LOMG more common in men [4,5]. Very late-onset MG is a category for individuals over 65 years old [5]. This subset group has an increased prevalence of comorbidities. 

Acetylcholine receptor (AChR) antibodies are present in 85% of cases (AChR positive), muscle-specific tyrosine kinase (MuSK) in 6%, and lipoprotein receptor-related protein 4 (LRP4) in 2%. Seronegative MG (SNMG) accounts for fewer than 10% of cases. MG typically presents with diplopia or lid ptosis in 15% of cases, and when the symptoms are confined to the eyes, it is designated as ocular MG (OMG). Ninety percent of these cases will generalize to generalized MG (gMG) within two years [4]. A myasthenic crisis occurs if the respiratory muscles are affected, which can be life-threatening and may require inpatient intensive care management. MG treatment depends on factors such as serological status, disease severity, muscle weakness, medication efficacy, patient age, and medical comorbidities [2].

Conventional MG management includes the use of acetylcholinesterase inhibitors (e.g., pyridostigmine) for acute symptomatic relief. Disease-modifying immunosuppressive agents, which have a delayed onset of action (up to six months), include corticosteroids, azathioprine, mycophenolate mofetil, and cyclosporine. Immunomodulatory therapies, which act more rapidly (within weeks), include intravenous immunoglobulin (IVIG) infusion and plasmapheresis [2,6]. Immunoglobulin may also be administered subcutaneously for maintenance therapy. Thymectomy may be considered in select patients to optimize treatment response.

Intravenous efgartigimod alfa (EA) was approved in December 2021 for the treatment of AChR antibody-positive gMG [6]. It is administered in treatment cycles as an intravenous infusion once weekly for four weeks. If needed, another cycle is administered at least four weeks apart. The response is measured by the MG Activities of Daily Living Scale (MG-ADL), an eight-item validated assessment tool designed to measure deficits resulting from MG [7]. Scores range from 0 to 24, with higher scores indicating greater impairment. A two-point improvement within the first four weeks was considered indicative of effective treatment in the ADEPT trial [2,6].

## Case presentation

A 90-year-old woman with colorectal cancer undergoing chemotherapy treatment was referred to our clinic by neuro-ophthalmology for gMG. Her serology confirmed AChR positivity. She presented with bilateral eyelid ptosis, causing vision obstruction, but did not have diplopia. She also experienced difficulty expelling mouthwash due to facial muscle weakness and noticed a decrease in her voice strength. She denied nasal regurgitation of fluids. For years, she had used her arms to rise from a low chair due to weakness, and she required an assistive device to walk safely. On examination, her left eye was completely closed, and her right eye was 80% closed. The strength of bilateral eyelid closure was 4/5 on the Medical Research Council (MRC) Scale. She had full extraocular movements bilaterally. Neck flexion was weak (4/5), while neck extension strength was normal (5/5). Jaw opening strength was normal, and there was no glossal triple furrow sign or atrophy. She had no respiratory difficulty. She used her arms to rise from a chair, compensating for hip flexion weakness (4/5). Her smile was symmetrical. She could only transiently puff out her cheeks but could easily pucker her lips. No fatigable weakness was observed in shoulder abduction. Weakness (4/5) was noted in the bilateral hip flexors, right-hand finger extensors, and left wrist extensors. Bilateral long finger flexor strength was normal. Left ankle dorsiflexion was weak (4/5), and she had a fused right ankle. A CT scan of the chest was normal. Her calculated MG-ADL score was 7 (normal, 0).

**Figure 1:**
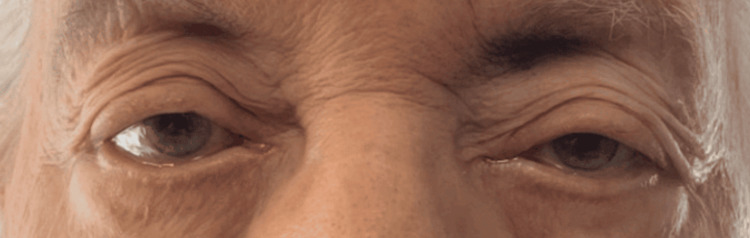
After two weeks of treatment, residual left ptosis was relieved, improving visual obstruction. *The picture was obtained with permission from the patient.

**Figure 2:**
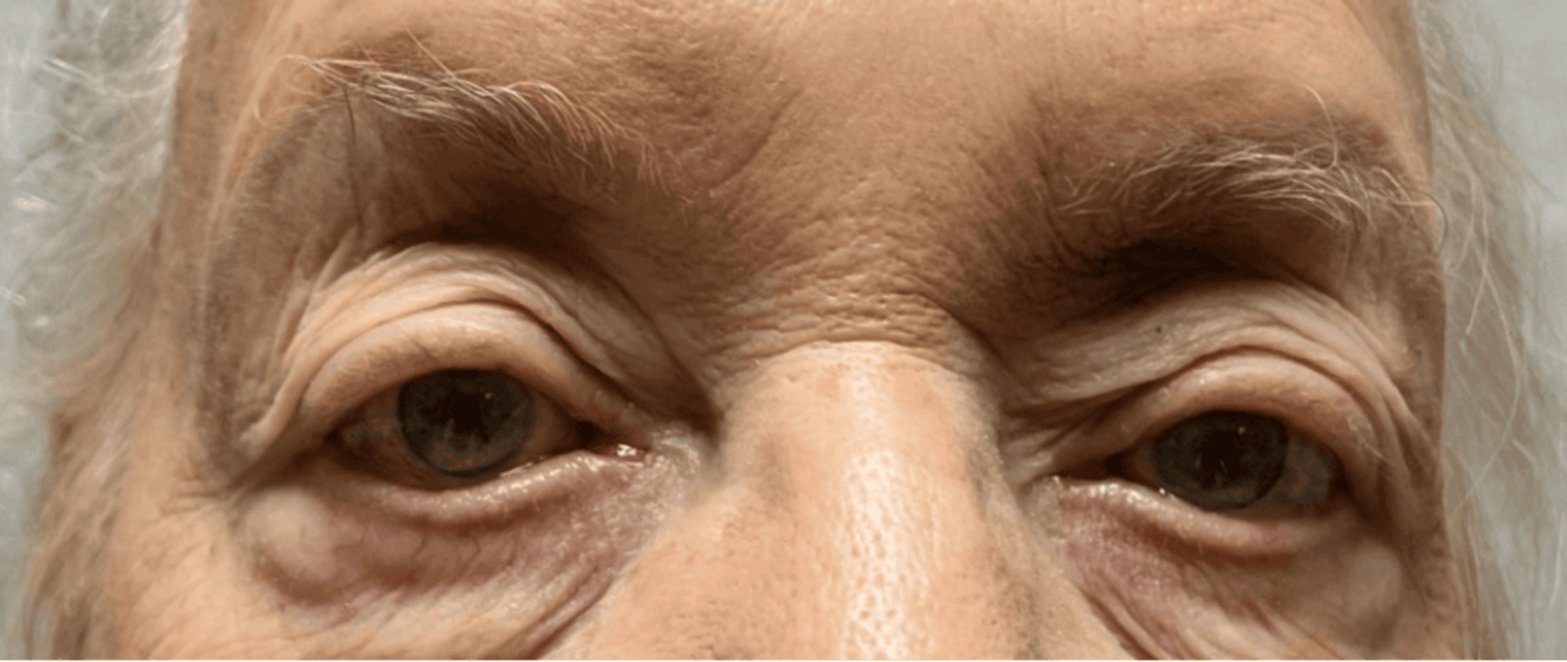
After one cycle of treatment (four weeks). *The picture was obtained with permission from the patient.

Although the initial presentation was not captured, significant improvement in ptosis was observed by the end of the second week of treatment (Figure 1). By the fourth week, ptosis had nearly resolved completely (Figure 2). Additionally, her voice showed noticeable improvement. She tolerated the infusions well. Her MG-ADL score improved from 7 (pretreatment score) to 4 after one cycle of treatment. The patient expressed satisfaction with the treatment. She has been on EA for 10 months for symptom relief without experiencing any adverse effects.

## Discussion

**Figure 3:**
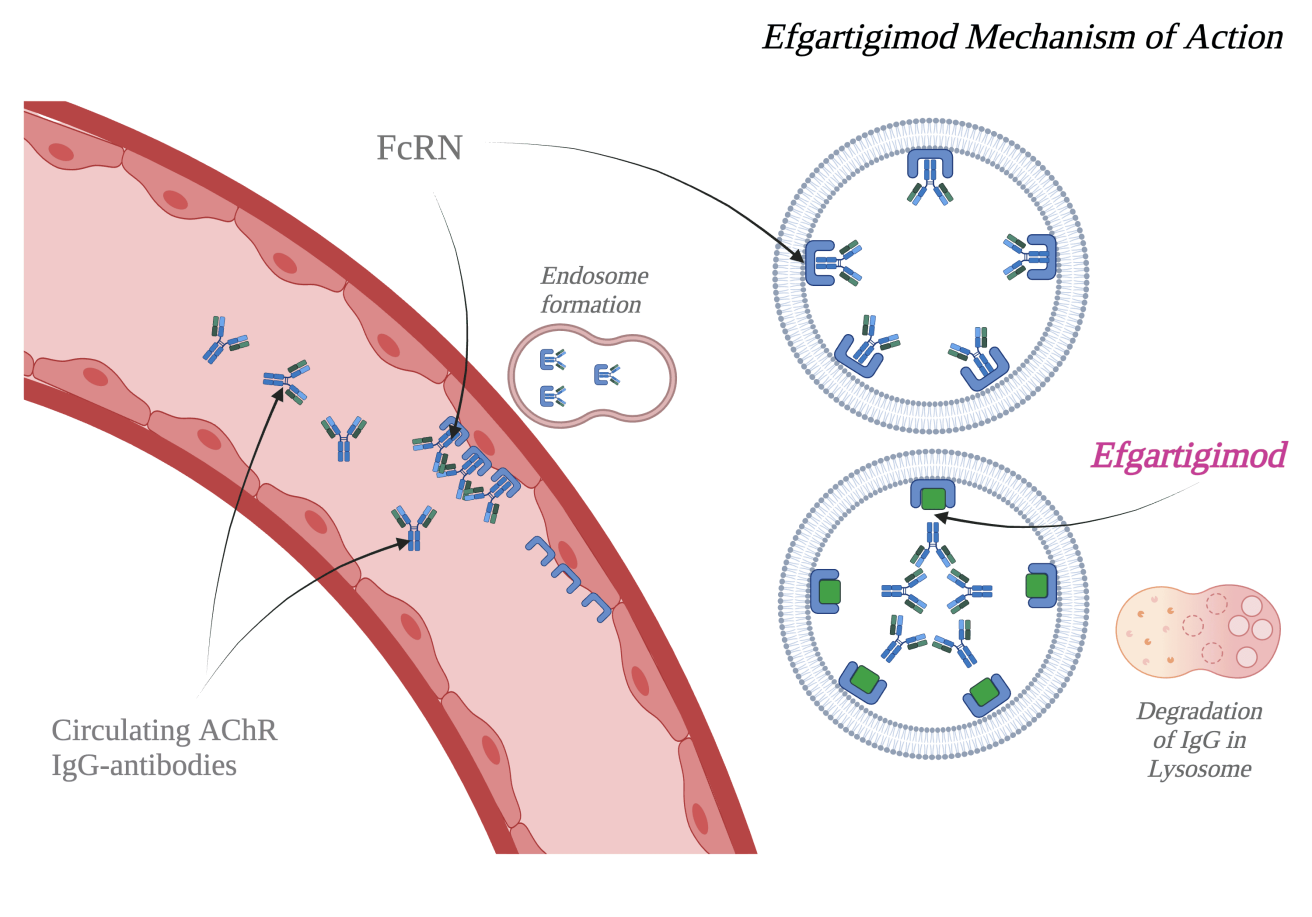
Efgartigimod alfa decreases circulating pathogenic IgG by blocking the release from lysosomal degradation. Image credit: Octavio Carranza Renteria. Made in BioRender (https://www.biorender.com).

MG is a chronic autoimmune disorder that targets the neuromuscular junction, causing muscle weakness that is typically worse at the end of the day. Antibody-mediated attacks on the postsynaptic neuromuscular junction (Figure 3) result in muscle weakness in MG. First-line treatments for MG are effective but may have multiple adverse effects and delayed onset. These treatments may not be optimal for the elderly, particularly those with significant comorbidities [8]. In our patient with colorectal cancer, we did not follow standard treatment guidelines for gMG for the following reasons: the delayed onset of immunosuppressants (such as mycophenolate or azathioprine), the multiple adverse effects of steroids (such as hypertension, hyperglycemia, and immunosuppression), and the potential worsening of cancer with anti-CD20 therapies or the increased risk of coagulopathy with IVIG. Additionally, pyridostigmine could exacerbate her preexisting diarrhea, and complement inhibitor medications were not suitable due to the requirement for additional pre-infusion vaccinations. We selected EA treatment for its early onset of benefit, less potent immunosuppression, favorable side effect profile, and unique mechanism of action. EA is a neonatal Fc receptor antagonist that decreases circulating levels of pathogenic IgG antibodies through increased lysosomal degradation (Figure 3) [6]. It is administered in treatment cycles as an intravenous infusion at 10 mg/kg (with a maximum dose of 1,200 mg IV) once weekly for four weeks. If needed, another cycle can be administered, spaced at least four weeks apart. EA has demonstrated treatment efficacy and safety in patients with gMG [2,6]. Its main side effects include a transient decrease in white blood cells, headache, and increased incidence of respiratory and urinary tract infections. Less than 10% of patients report paresthesias, myalgias, and minor hypersensitivity reactions such as rash and dyspnea, which typically occur within one hour to three weeks after treatment. During post-marketing surveillance, cases of anaphylaxis and angioedema have been reported, although these are rare. There are no contraindications other than hypersensitivity to the medication [6]. In our patient, EA was effective, fast, and well tolerated, showing improvements in visual disability and voice strength within two weeks. The International Consensus Guidance for the Management of MG was last updated in 2020 [9], and with the addition of new therapies, these guidelines need to be reviewed [2,3,6].

## Conclusions

LOMG patients with multiple comorbidities may experience worsening symptoms with traditional therapies. EA, a neonatal Fc receptor antagonist, offers a low side effect profile and faster onset of action, making it a potential first-line treatment for this group. We present a case of a 90-year-old woman with severe ocular symptoms from gMG and underlying cancer who was successfully treated with EA intravenously as a first-line agent instead of following standard treatment guidelines.

In our case report, the results demonstrate that EA led to rapid improvements in visual disability, voice strength, and quality of life scores. Furthermore, this case highlights that EA is a well-tolerated medication that provides symptom relief without the unwanted side effects associated with conventional therapies. Given these findings, current treatment guidelines need to be reconsidered in light of therapeutic advances.
